# PARP inhibitor olaparib induces DNA damage and acts as a drug sensitizer in an in vitro model of canine hematopoietic cancer

**DOI:** 10.1186/s12917-025-04880-z

**Published:** 2025-07-05

**Authors:** Jayson Cagadas Pasaol, Ewa Dejnaka, Greta Mucignat, Joanna Bajzert, Marta Henklewska, Bożena Obmińska-Mrukowicz, Mery Giantin, Marianna Pauletto, Christopher Zdyrski, Mauro Dacasto, Aleksandra Pawlak

**Affiliations:** 1 Department of Pharmacology and Toxicology, Faculty of Veterinary Medicine, Wroclaw University of Environmental and Life Sciences, Norwida 31, Wroclaw, 50-375 Poland; 2 Department of Comparative Biomedicine and Food Science, Division of Veterinary Pharmacology and Toxicology, University of Padua, Legnaro, 35020 Italy; 3 Department of Immunology, Pathophysiology and Veterinary Preventive Medicine, Faculty of Veterinary Medicine, Wroclaw University of Environmental and Life Sciences, Norwida 31, Wroclaw, 50-375 Poland; 4 Precision One Health Initiative, College of Veterinary Medicine, University of Georgia, 220 Riverbend Road, Athens, GA 30602 USA

**Keywords:** Canine lymphoma, Canine leukemia, Targeted therapy, DNA repair, Doxorubicin synergy, Mutational analysis

## Abstract

**Background:**

The introduction of genetic tests based on next-generation sequencing techniques into veterinary cancer diagnostics provides information on molecularly targeted therapies useful for dogs. However, there is still a lack of in vitro studies describing the effect and mechanism of action of such anti-cancer drugs in companion animals. Our study aimed to demonstrate in vitro activity of a commonly used PARP inhibitor, olaparib, in canine lymphoma and leukemia cells as well as to indicate its potential uses in anti-cancer therapy based on the mutational status of DNA damage related genes. Canine lymphoma and leukemia cell lines were incubated with olaparib alone and in combination with doxorubicin, and the impact of a single drug and combinations on cell viability, proliferation, induction of apoptosis, and DNA damage were assessed.

**Results:**

The study showed that olaparib acts as a single agent, inhibiting the metabolic activity of canine lymphoma (CLBL-1, CNK-89) and leukemia (CLB70, GL-1) cells, affecting cell proliferation rates and causing DNA damage. In the tested cells, olaparib also worked as a chemosensitizer, due to its ability to potentiate cytotoxic effects of doxorubicin. Finally, RNA-seq data identify various mutational burden differences in genes involved in the DNA damage response in CLBL-1 and GL-1 cell lines that may explain the observed in vitro sensitivity differences to olaparib.

**Conclusions:**

Olaparib may be an interesting oral therapy alternative to classic chemotherapy or adjuvant option in dogs with hematopoietic cancer with known DNA repair disorders.

**Supplementary Information:**

The online version contains supplementary material available at 10.1186/s12917-025-04880-z.

## Background

The search for innovative targeted anti-cancer treatments is critical for modern oncology. As access to genetic tests for dogs becomes widespread, physicians will gather more data providing indications for the use of novel medications targeting specific molecules. Interestingly, common availability of modern molecular methods for cancer diagnosis in animals has significantly outpaced the rate of research on the use of molecularly targeted drugs, thus creating a gap between information about dedicated treatment and the actual possibility of using specific substances in animals. An example may be the use of poly (ADP-ribose) polymerase (PARP) inhibitors (PARPis), drugs with already proven effectiveness in humans, for treatment of patients with DNA damage repair-deficient cancers, mainly with Breast Cancer Associated 1 and 2 (BRCA1 and BRCA2) mutations [1].

PARP1 plays a crucial role in repairing DNA damage. Following DNA insult, PARP1 is rapidly recruited to single-strand breaks (SSBs) and double-strand breaks (DSBs) in DNA. Subsequently, BRCA1 and BRCA2 are recruited as they regulate the major pathway for DSB repair, homologous recombination (HR) [1]. Pharmacological inhibition of PARP1 leads to DNA damage and, in the absence of functional *BRCA1* and *BRCA2* genes, to critical levels of genomic instability provoking cell death [2]. Cell death observed after PARPi treatment of HR-deficient cells is based on synthetic lethality. This occurs when mutation-induced dysfunctions in two genes do not affect cell viability separately, but simultaneous disorders in both genes lead to cell death [3]. Thanks to this effect, PARPis quickly became a novel class of anti-cancer drugs, first demonstrating efficiency in treating HR-deficient tumors with a *BRCA1* and *BRCA2* mutation [4]. Interestingly, patients with genetic HR dysfunction are not the only candidates for PARPi treatment. Some sporadic cancers are also characterized by HR deficiency but without *BRCA1-2* mutations. The broad range of DNA substrates and various processes targeted by PARPis imply that PARPis may also reduce survival of other DNA repair-deficient cells [5], a phenomenon referred to as BRCAness [6].This observation supports current clinical trials of PARPis in patients with other HR-deficient, *BRCA*-dependent cancers [7].

Despite the success of PARPis in human oncology, these drugs are not extensively researched in veterinary medicine [8, 9]. Considering the current wide indications for PARPis and increasing data on HR disorders in various dog cancers [10], we decided to investigate the activity of the first and best-studied PARPi, olaparib, in canine hematopoietic cancers. We assessed the antiproliferative and proapoptotic effect of olaparib, and its ability to induce DNA damage. Additionally, we investigated the synergistic effect of olaparib and doxorubicin to determine their potential utility in chemotherapy for hematological malignancies. Finally, we analyzed the mutational profile of key genes involved in the DNA damage response to assess their impact on olaparib sensitivity in vitro.

## Results

### Olaparib shows activity as a single agent in canine lymphoma/leukemia cells

#### Inhibition of cell metabolic activity

**Fig. 1:**
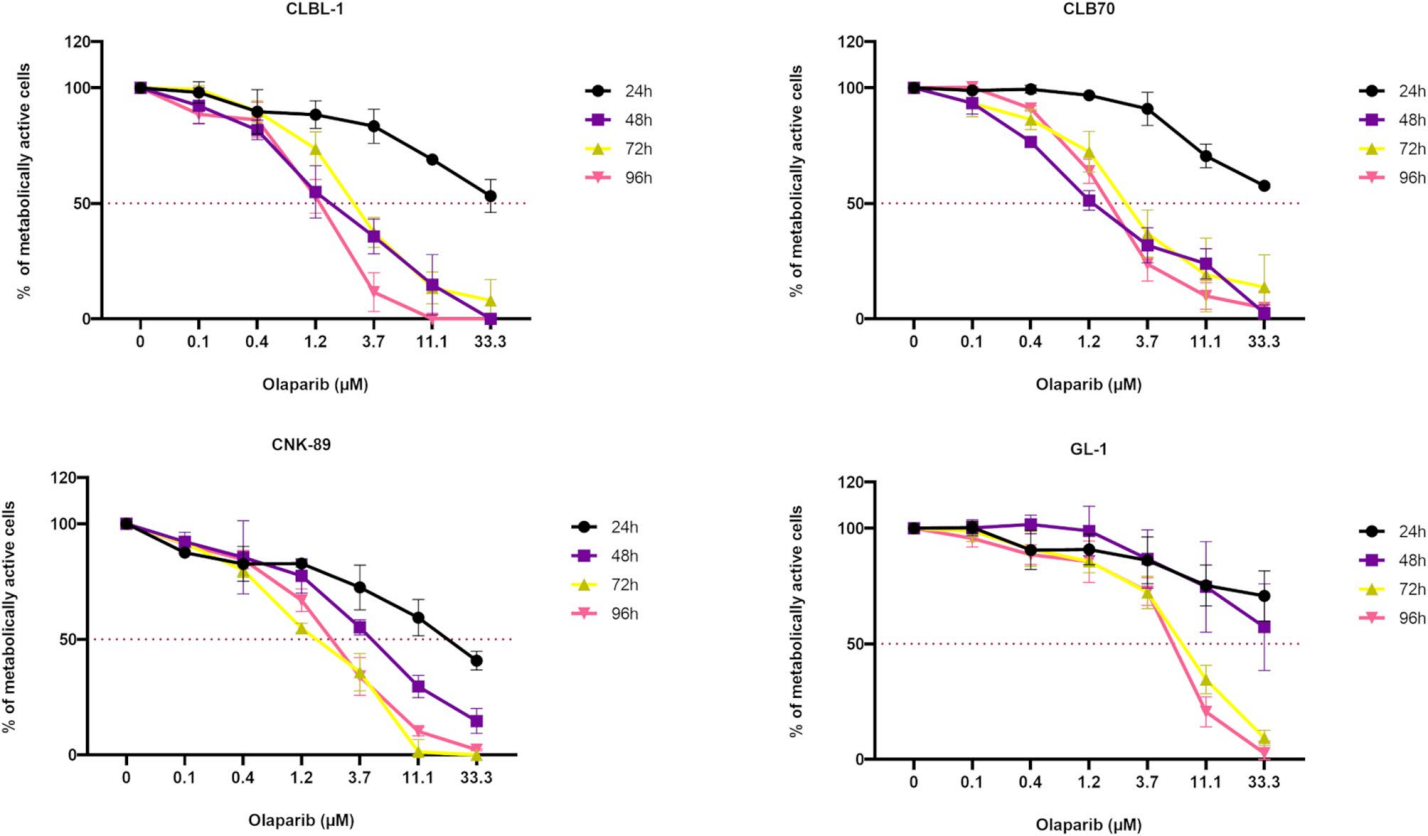
Concentration-dependent curves displaying the effects of olaparib. Metabolic activity (measured by MTT assay) of CLBL-1, CLB70, CNK-89, and GL-1 cell lines after 24, 48, 72, and 96 h of incubation

To check if PARPis may be a potential therapeutic option for dogs with lymphoma or leukemia, we first investigated the effect of olaparib on the metabolic activity of established canine cancer cell lines using the MTT (3-[4,5-dimethylthiazol-2-yl]-2,5-diphenyl tetrazolium bromide) assay. Obtained data indicated that olaparib alone inhibits the cell metabolic activity of all lymphoma/leukemia cell lines used in our study in both concentration- and time-dependent manners. Concentration-dependent curves presenting the effects of olaparib on the metabolic activity of the tested cell lines are shown in Fig. 1.

We identified some differences in the sensitivity of individual cell lines to olaparib, with the GL-1 cell line being less susceptible compared to the others. As expected, the cytotoxic effect became more evident with prolonged incubation, reaching the concentration that inhibited cell metabolic activity by 50% (IC_50_ value) below 3 µM after 72 h for all the sensitive cell lines. This concentration is much lower than the maximum one achievable in human patients with solid tumors treated with 10–400 mg olaparib twice daily [11]. A comparison of IC_50_ values for each cell line is presented in Table 1.

**Table 1:** The cytotoxic effects of Olaparib after 48, 72, and 96 h of incubation on the canine lymphoma/leukemia cell lines, expressed as IC_50_ values. The results are presented as mean ± standard deviation (SD) of 3 independent experiments, each performed in triplicates. Statistical differences were analyzed using a one-way ANOVA followed by the tukey’s multiple comparison test. Values without common letters (a, b, c) in the superscript differ statistically (*P* <.05)

IC_50_ (µM) values after olaparib treatment of canine lymphoma/leukemia cell lines				
	CLBL-1	CLB70	CNK-89	GL-1
48 h	5.60^a^ ± 1.69	4.82^a^ ± 0.84	4.29^a^ ± 0.14	> 33.3^b^
72 h	2.68^a^ ± 0.81	3.03^a^ ± 1.37	2.03^b^ ± 0.28	6.83^c^ ± 1.57
96 h	1.2^a^ ± 0.17	2.09^a^ ± 1.44	1.50^a^ ± 0.63	5.57^b^ ± 1.34

#### Antiproliferative effect

**Fig. 2:**
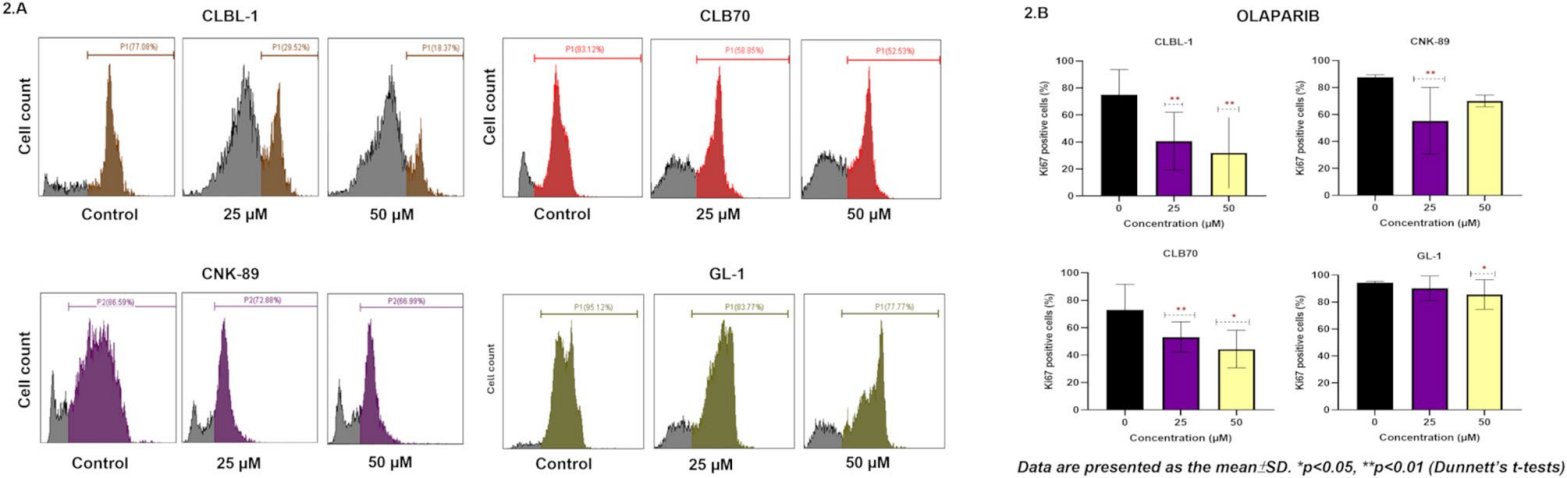
Histograms presenting percentages of proliferating (Ki-67 positive cells). Cells after 48-hour treatment with 25 and 50 µM of olaparib with reference to the control (**A**). Graphs displaying the statistical analysis of the results (**B**). Values are presented as means ± standard deviation (SD) of three independent experiments

To investigate the antiproliferative effect of olaparib on canine lymphoma/leukemia cells, the expression of Ki-67 (a common proliferation marker) was determined in cells treated with olaparib for 48 h. The percentage of Ki-67 positive cells (as compared to the untreated control) decreased significantly (*P* <.05) after incubation with 25 and 50 µM of olaparib in all tested cell lines (Fig. 2). The strongest effect was observed for the CLBL-1 cell line, where Ki-67 expression dropped below 20% after 48 h of incubation with 50 µM of olaparib, indicating a decrease in the cell proliferation potential.

#### DNA damage induction

**Fig. 3:**
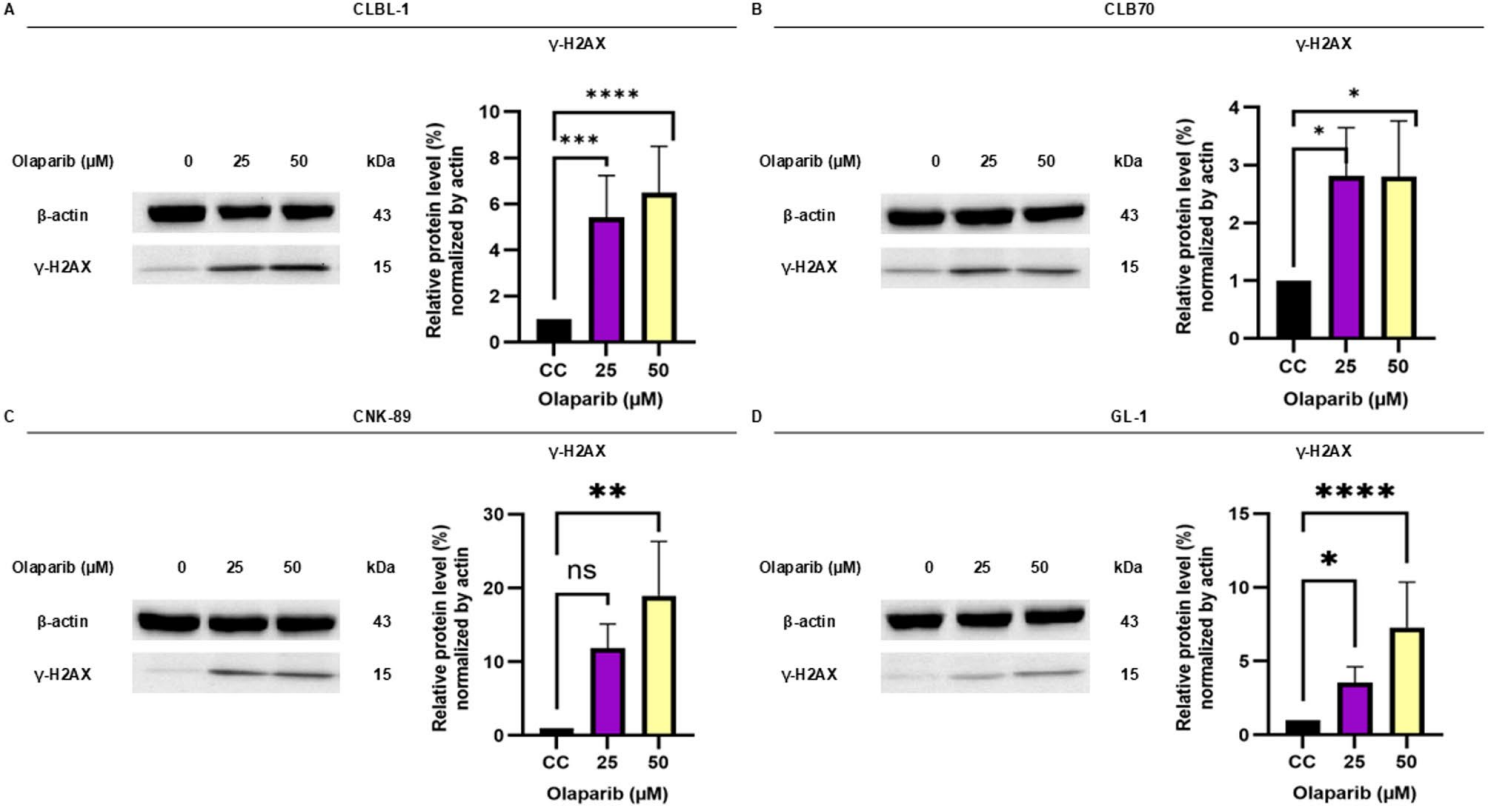
Western blot analysis for phosphorylated histone H2A.X. The CLBL-1, CLB70, CNK-89, and GL-1 (**A-D**) cell lines after 24 h of incubation with different concentrations of olaparib (25 and 50 µM). Quantification was performed by normalizing the expression level of the protein of interest to the expression level of the loading control, β-actin. Mean and standard deviations were calculated based on three independent experiments. The asterisks (*) are used to represent comparative statistically significant results. *P <.05; **P <.01; ***P <.001; ****P <.0001; ns– not significant (*P* >.05)

Having confirmed that olaparib, as a single agent, exerts cytotoxic and antiproliferative effects on canine lymphoma/leukemia cells, we decided to investigate if it also causes DNA damage in sensitive cells. We sought to determine if and after what incubation time olaparib causes DNA damage to accumulate in the cell and can be detected as phosphorylation of histone H2A.X on serine 139, (γH2A.X), a verified and common marker of DNA damage. The results of the histone H2A.X phosphorylation analysis using the Western blot technique are shown in Fig. 3. Olaparib indeed induces the formation of DNA damage, even after 24 h of incubation, also in the cells with limited sensitivity to olaparib (see MTT results, Fig. 1). This result clearly shows that the accumulation of DNA damage in the cells disturbs cell proliferation.

### Olaparib acts as a chemosensitizer due to its ability to potentiate the cytotoxic effects of doxorubicin– a commonly used cytostatic drug for lymphoma/leukemia therapy

#### Olaparib– doxorubicin combinatory effects

**Fig. 4:**
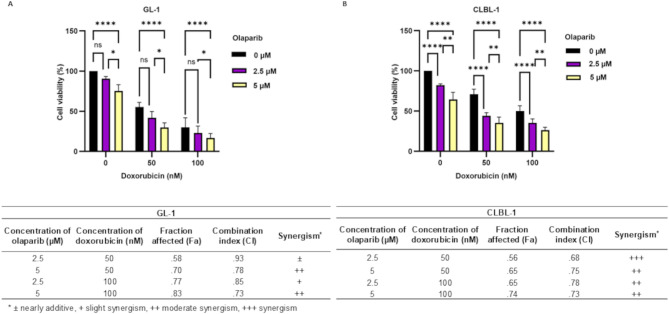
Analysis of synergistic effect of olaparib with doxorubicin. Cell metabolic activity after incubation with olaparib (96 h), doxorubicin (48 h), or preincubation with olaparib for 48 h and following exposure to the combination of doxorubicin and olaparib for the next 48 h (MTT assay) (**A**). Combination indexes (CI) of olaparib and doxorubicin in GL-1 and CLBL-1 cells (**B**). ± indicates nearly additive, + slight synergism, ++ moderate synergism, +++ synergism. The asterisks (*) are used to represent comparative statistically significant results. *P <.05; **P <.01; ****P <.0001; ns– not significant (*P* >.05)

To evaluate the potential benefit of adding olaparib to a conventional anti-cancer treatment in dogs, the effects of a combined exposure of lymphoma/leukemia cells to olaparib and doxorubicin were analyzed. The resulting CI (Combination Index) values (Fig. 4B) indicated that doxorubicin and olaparib showed a moderate synergy in most combinations in the CLBL-1 cell line, and slight to moderate synergy in the GL-1 cell line, except for the combination of the lowest concentrations, where the effect was rather additive. It is worth mentioning, that although the synergy was more evident in the CLBL-1 cell line, especially at the lowest concentrations of both drugs, it increased in the GL-1 cell line with increasing drug concentrations, which was also associated with higher fraction affected (Fa) values. Most importantly, as shown in Fig. 4A, combinations of both agents at concentrations affecting at least 50% of the cells in monotherapy decreased cell metabolic activity by more than 70% at the highest olaparib concentration. This effect was again more pronounced in the CLBL-1 cells (Fig. 4B). The study indicated that olaparib can also be used as a drug that sensitizes cells to the action of doxorubicin.

### Genes involved in DNA repair are mutated in the CLBL-1 and GL-1 cell lines

#### Identification of exonic variants from RNA-seq data

The complete list of detected SNVs (single-nucleotide variants) and INDELs (small insertions or deletions) is reported in Supplementary Table 1. In particular, INDELs and SNVs were found on 3’/5’ UTRs together with 11 and 14 synonymous SNVs in CLBL-1 and GL-1 cells, respectively. The Transeq tool was used to examine each INDEL, and premature stop codons were never identified. A focus on missense SNVs was also provided in Table 2, which reports for each SNV the coding sequence (CDS) position, the exon, the frequency of the alternate allele, and the VEP/Fido-SNP effect prediction.

**Table 2:** Missense SNVs identified in DNA-damage response genes of CLBL-1 and GL-1 cell lines

	Cell line	CDS position	Exon	Frequency of the alternate allele	VEP prediction	Fido-SNP prediction
*BRCA1*	GL-1	c.604 A > G	9	HE	Tolerated	Benign
		c.715G > A	10	HE	Tolerated	Benign
		c.1329 A > C	10	HE	Deleterious	Pathogenic
		c.3619 A > G	10	HE	Tolerated	Benign
		c.5186G > A	13	HE	Tolerated	Benign
*BRCA2*	CLBL-1	c.428 A > G^a^	5	HE	Tolerated	Pathogenic
		c.1158T > G^a^	10	HO	Tolerated	Benign
		c.2401 A > C	11	HE	Deleterious	Benign
		c.4304 A > G^a^	11	HE	Tolerated	Benign
	GL-1	c.428 A > G^a^	5	HE	Tolerated	Pathogenic
		c.1158T > G^a^	10	HE	Tolerated	Benign
		c.4304 A > G^a^	11	HE	Tolerated	Benign
		c.6686G > T	11	HE	Tolerated	Benign
*TP53*	CLBL-1	c.374 C > T	5	HE	Deleterious	Pathogenic
		c.764G > A	8	HE	Tolerated*	Pathogenic
	GL-1	c.709 C > T	7	HO	Deleterious	Benign
*ATM*	CLBL-1	c.3431 A > T^a^	25	HE	Tolerated	Benign
		c.5267T > C^a^	36	HO	Tolerated	Benign
	GL-1	c.3431 A > T^a^	25	HO	Tolerated	Benign
		c.5267T > C^a^	36	HO	Tolerated	Benign
*ATR*	CLBL-1	c.539G > A	4	HO	Tolerated**	Pathogenic

^a^SNVs shared by CLBL-1 and GL-1 cell lines; *SIFT(Sorting Intolerant From Tolerant) = 0.09; **SIFT = 0.05; HE: heterozygous; HO: homozygous

Among the genes considered for the present analysis, *TP53* appeared to be the most biologically affected; it showed two heterozygous adverse SNVs (c.374 C > T; c.764G > A) in CLBL-1 cells and a homozygous one (c.709 C > T) in GL-1 cells.

*BRCA1* was mutated in the GL-1 cell line only; among the 5 missense SNVs, only c.1329 A > C was predicted as deleterious/pathogenic by VEP and Fido-SNP tools. Both cell lines carried mutations on the *BRCA2* transcript, but the CLBL-1 cell line seemed to be the most affected one; three SNVs were in common, while a deleterious SNV (c.2401 A > C) and a deletion (c.6916_6918del) were specifically identified in CLBL-1 cells. It is worth noting that some SNVs shared by GL-1 and CLBL-1 cells had a different rate of expression, suggesting a differential impact on the two cell lines; as an example, c.9995_9996insAAA and c.1158T > G in *BRCA2* were homozygous in CLBL-1 and heterozygous in GL-1 cells (Supplementary Table 1); conversely, c.3431 A > T in *ATM* was homozygous in GL-1 and heterozygous in CLBL-1 cells.

*ATR* carried a common deletion (c.1550_1561del) in both cell lines. Moreover, in the CLBL-1 cell line, a homozygous missense SNV (c.539G > A) was predicted pathogenic by Fido-SNP and tolerated at the limit of tolerance (SIFT = 0.05) by VEP. Interestingly, for *RAD51*, a common silent SNV predicted pathogenic by Fido-SNP was noted. Likewise, c.1050G > A (for GL-1 cells) and c.1359G > A (for CLBL-1 cells) in the *ATM* gene were predicted pathogenic even if synonymous. In the *STK11* coding sequence, only one common synonymous SNV was observed, showing a different frequency of alternate alleles between the two cell lines; conversely, no variants were found in *CHEK1* and *CHEK2* transcripts. All these data are presented in Supplementary Table 1.

## Discussion

Over the last few decades, biotechnological breakthroughs have led to the identification of complex and unique biologic features associated with carcinogenesis and thus cancer treatment. Genomic analysis, especially NGS has revealed a complex truth about cancer, necessitating a transition in therapy from tumor type-centered to gene-directed personalized treatment based on biomarkers [12]. Nowadays, veterinary oncology is transitioning to a genomics-based strategy for informing treatment of the patient. Individual mutations in cancer-bearing dogs are better understood, which encourages the use of genomics for diagnosis, prognosis, and even treatment of canine patients. Such progress in research is also crucial for the translational importance of veterinary oncology. Due to numerous similarities between canine and human cancers, genetic data from canine tumors can be exploited to find therapeutic response signals to inform and guide human drug development [13]. On the other hand, an improved understanding of cancer’s molecular foundation has profoundly transformed the therapy landscape for veterinary oncology patients. For example, single gene-based assays have been available for years for diagnosing *KIT* mutations in mast cell tumors (MCTs), while assays for *BRAF* mutations in urothelial carcinomas have just been identified [14, 15]. An increasing number of companies and tools enabling genomic diagnostic testing are providing a powerful data source for biomarker discovery and cancer profiling for dogs. Examples of such tools are: are SearchLight DNA cancer genomic diagnostic assay [16] and The FidoCure^®^ Precision Medicine Platform [17].

The SearchLight DNA assay is a cancer NGS gene panel for dogs that uses hybrid capture-based enrichment of 120 canine cancer-related genes to detect single nucleotide variants (SNVs), small insertions or deletions (INDELs), copy number variants (CNVs), and internal tandem duplications (ITDs) using tumor-only sequencing [16]. This panel identifies mutated genes that inform sensitivity to PARPis. These genes are primarily those involved in the DNA damage repair process, mainly related to the HR repair pathway: *ATM*,* CHEK2*,* BRCA1*,* BRCA2*,* FANCL*,* PALB2*, and *CDK12* [10]. Chon et al. 2023 showed clinical and genomic data from 134 cancer-bearing dogs profiled by the SearchLight DNA assay to identify mutation-level predictive relationships and evaluate the influence of therapy decisions based on patient-specific mutations [18]. The indication for olaparib treatment was found in dogs with hepatic sarcoma, melanoma, osteosarcoma, renal carcinoma and sarcoma, soft tissue sarcoma, squamous cell carcinoma, and thymic carcinoma. The study showed improved survival in dogs receiving genomically informed olaparib treatment. Despite a lack of research on the mechanism of action, cytotoxicity, and effectiveness of olaparib in the abovementioned types of cancer in dogs, this drug was used and proved to be effective. It was administered at a dose ranging from 1.4 to 3 mg/kg per day [16]. In another study, using the FidoCure^®^ Precision Medicine Platform, the authors analyzed the prognostic effects of treatments based on 5 tumor genomic alterations in the *TP53*,* PIK3CA*,* NRAS*,* ATM*, and *KIT* genes. Based on the NGS results, olaparib was recommended for 59 patients with *ATM* mutations.

In our study, we used RNA-seq data to identify various mutational burden differences in genes involved in the DNA damage response in CLBL-1 and GL-1 cell lines that may explain the differential in vitro sensitivity to olaparib. Focus was placed on variants that could alter the amino acid sequence (missense SNVs and INDELs) and in target genes showing the most significant results. However, it should be noted that even synonymous SNVs, albeit not fully taken into consideration, might also sometimes impact protein activity, as demonstrated by Kimchi-Sarfaty and colleagues in the case of mdr1 [19]. In the presented study, we identified missense SNVs in selected DNA damage response genes in both tested canine lymphoma/leukemia cell lines. We started our analysis with the two most important genes associated with sensitivity to PARPis: *BRCA1* and *BRCA2*. Both genes’ germline mutations have been linked to an increased risk of several human malignancies, including breast and ovarian cancers. The repair of DNA DSBs by homologous recombination depends on *BRCA1/2*; specifically, BRCA1 is fundamental for the recruitment of BRCA2 to the site of DSBs, and in turn, BRCA2 is essential for the recruitment of RAD51 [20]. All *BRCA1* missense and synonymous SNVs we observed in the GL-1 cells were also found in canine normal and tumor mammary samples [21]. Additionally, the same authors confirmed all the *BRCA2* variants found in our study, except for c.6686G > T (p.Cys2229Phe). In the case of *BRCA2*, most of the work was focused on variants located in exon 11, a region that is largely conserved across different species. In fact, BRC repeats that are involved in RAD51 binding are present in this exon and variations that impact RAD51 binding are crucial and may hinder HR-mediated DNA repair [22]. Interestingly, both GL-1 and CLBL-1 cell lines disclosed specific SNVs in this region. The c.4304 A > G (p.Lys1435Arg) was already reported in canine mammary tumors and it is precisely located in BRC3 [23]. The same SNV was previously reported together with c.2401 A > C (p.Lys801Gln), being the most frequent SNV registered in both canine mammary tumors and normal mammary tissue [24, 25]. Moreover, both were predicted as deleterious [26]. Another finding concerning *BRCA2* is the insertion c.9995_9996insAAA in exon 27, which is heterozygous in GL-1 cells and homozygous in CLBL-1 cells. Exon 27 encodes for the nuclear localization signals, one of RAD51 binding sites, and a cyclin-dependent kinase (CDK) phosphorylation site [27, 28]. This insertion was previously detected in dogs, but its pathogenicity is still controversial [23, 25, 29]. However, it is known that it enhances the nuclear localization of the BRCA2 protein, possibly boosting the DNA damage repair machinery [30]. Considering that both cell lines carry deleterious mutations on *BRCA1* and/or *BRCA2* genes, it’s quite challenging to affirm that this could be the main or unique cause of a differential response to olaparib in vitro. According to the authors, differences in the *TP53* and *ATR* genes, and not *BRCA1/2*, may be the primary source of the observed differential sensitivity.

*TP53* is a tumor suppressor gene, also known as the “guardian of the genome”. It is sensitized by DNA damage and leads to cell cycle arrest in the G1 phase [31]. *ATR* responds to a wide range of genotoxic stimuli and is mainly activated by DNA single-strand breaks. Upon activation, ATR phosphorylates multiple downstream proteins, especially serine/threonine protein kinase CHK1, (CHK1; coded by *CHEK1*), which is a pathway that plays a crucial role in cell-cycle arrest [32–34]. In GL-1 we found one homozygous and in CLBL-1 we found two heterozygous deleterious SNVs in the *TP53* CDS. All of which were located in the DNA binding domain of P53 [35]. *TP53* mutations are common in cancerous cells and could impair the G1 checkpoint, avoiding cell cycle exit in case of DNA damage [36]. Therefore, *BRCA1/2* mutations often coexist with *TP53* ones [37–39] and mutations on *TP53* could induce resistance to PARPi treatment. In this respect, in human metastatic castration-resistant prostate cancer it has been described that the differential response to PARPi between *BRCA1* and *BRCA2* mutated cancers was due to the concomitant presence of a *TP53* mutation as well as the allelic representation [39, 40]. Nevertheless, this assumption cannot be applied to all cases, as each mutation and tumor could have specific mechanisms behind a therapeutic response. In our case, both cell lines have different defects in the *TP53* gene. It’s reasonable to think that a cell with an impaired ATM/CHK2/P53 axis must rely on other mechanisms to handle replicative stress, such as the previously described ATR/CHK1/WEE1 axis [36]. Compared to GL-1 cells, CLBL-1 cells are more likely to rely on the ATR axis according to previous studies [41], but interestingly *ATR* also showed deleterious mutations in this cell line.

Overall, since the mutational status of these two cell lines is lacking, except for the study of Das and colleagues (2019) [42] on CLBL-1 and other cancer cell lines, the present investigation partially fills the knowledge regarding these two cell models. Nevertheless, a limitation of the present study is that the detection of mutations was made exclusively on coding and *UTR* sequences of DNA damage response genes, thus intronic variants remain unknown. Moreover, it has already been described that the impact of a single variant might be compensated by other mechanisms [43–45]. Thus, more in-depth studies are needed to better define the potential causative association between mutations on DNA damage response genes and PARPi susceptibility.

The availability of genetic tests for dogs, as well as frequent indications for olaparib highlight the need to determine the effectiveness of PARP inhibition in canine cancer cells. In the present study, we showed that olaparib, even as a single agent, can negatively affect the metabolic activity and proliferation of canine lymphoma and leukemia cells. The cytotoxic effect of olaparib was particularly visible after at least 72 h of incubation, with the IC_50_ value of around 3 µM for sensitive lymphoma/leukemia cells. According to published data, the drug has similar potency against numerous human cancer lines, such as Ewing’s sarcoma, medulloblastoma, neuroblastoma, rhabdomyosarcoma, colon carcinoma, or osteosarcoma showing various potential applications [46, 47]. In canine lymphoma/leukemia cell lines, the antiproliferative effect of olaparib in vitro correlated with the expression of Ki-67, an important marker of cell proliferation. The effect was particularly visible for the CLBL-1 cell line, where Ki-67 levels dropped to 13% after 48 h of incubation with 50 µM olaparib. This effect was also observed in various cancer types with defects in their DNA repair pathways, for example in cancers with *BRCA2* mutations [48].

Sensitivity to PARP inhibition in canine leukemia/lymphoma cells is also evidenced by the presence of DNA damage. Our study found that olaparib causes DNA damage as soon as 24 h after incubation, even in the cells with low sensitivity to olaparib in the MTT test. This result indicates that DNA damage may be one of the reasons for the toxic effect of olaparib and suggests disruptions in the DNA repair pathway. Multiple studies show that olaparib as a single drug causes DNA damage in various cancer cell types, such as osteosarcoma [49], pancreatic cancer [50], prostatic cancer [51], lymphocytic and myeloid leukemia [52, 53] and lymphoma [54]. This validates the use of olaparib, even as a monotherapy, in cancers with suspected DNA repair dysfunction. At the same time, *ATM* mutations and ℽH2A.X expression profiles can serve as biomarkers of the response to PARPis, helping clinicians choose the optimal therapy for their animal patients [53]. Offering dog owners affordable oral therapy for their pets may be an alternative when they choose not to pursue intravenous treatment with classic therapeutic protocols.

Even greater expectations in the context of improving therapeutic effects are associated with the simultaneous use of PARPis and other drugs. Our study showed that adding olaparib to doxorubicin monotherapy brings better therapeutic results due to the synergistic effect of these two compounds (Fig. 4), giving an opportunity for the development of a less toxic therapy with increased anti-cancer effectiveness. The synergism demonstrated by these two substances used simultaneously will allow for the reduction of the therapeutic dose of doxorubicin compared to the dose used in monotherapy [55]. This is of particular importance because doxorubicin has a cardiotoxic effect, which limits the dose that can be used in the treatment of dogs (it is not recommended to exceed 240 mg/m^2^ cumulative dose) [56]. The possibility of using a lower dose of doxorubicin with preserved anti-cancer activity may be an opportunity for safe use of the drug also in dogs predisposed to the development of dilated cardiomyopathy (DCM), which show a five-fold higher prevalence of anthracycline-induced circulatory failure than other dogs [57]. Breeds predisposed to the development of DCM, and thus anthracycline-induced cardiomyopathy (AICM), include Dobermans, Great Danes, Rottweilers, and Boxers [57].

**Fig. 5:**
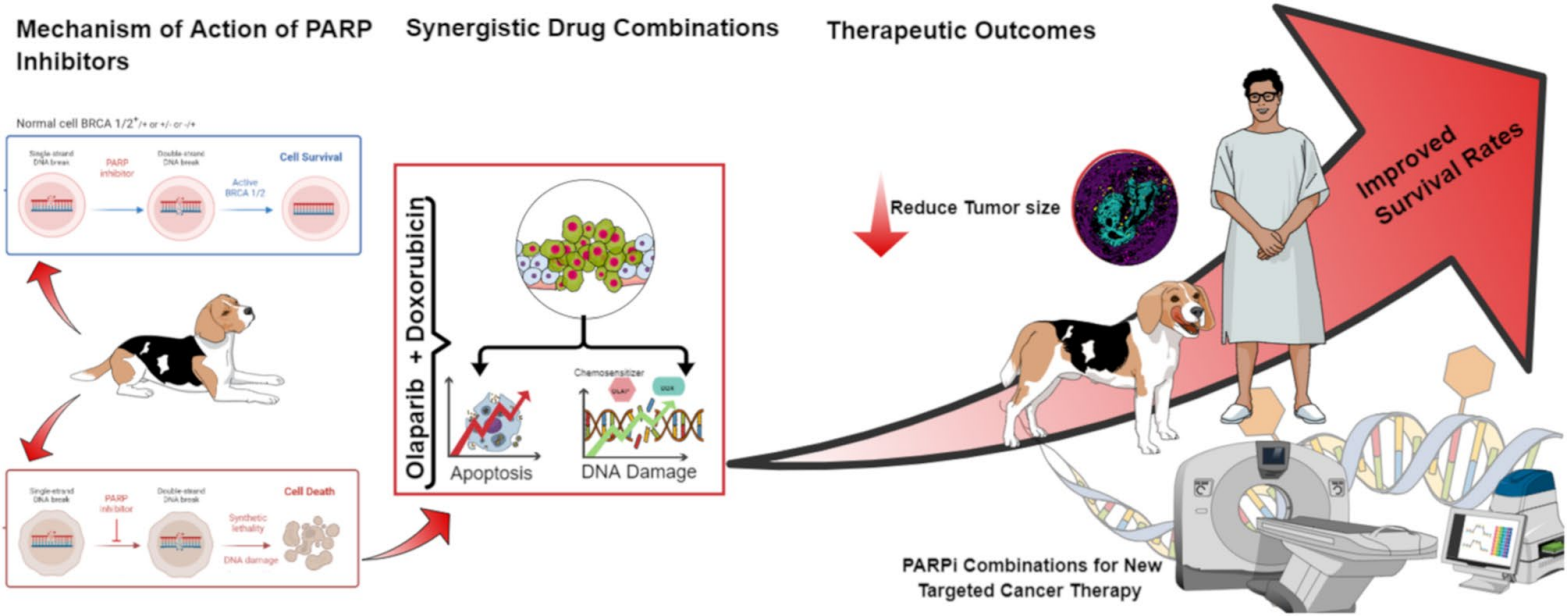
Schematic representations of the potential of PARPi in cancer therapy. Characterization of the molecular effects of PARPi combinations with additional drugs in various cancer types as a first step to develop new, molecular-based targeted therapeutic strategies in veterinary oncology

In osteosarcoma, the synergistic effect of the combined use of these two drugs was associated with increased apoptosis of cells as indicated by flow cytometry analysis and western blotting, which showed increased expression of cleaved PARP1, cleaved caspase 3, and BAX, and decreased expression of BCL2 [49]. In breast cancer it has been shown that one of the mechanisms through which PARP inhibition can chemosensitize cancer cells in vivo, is targeting Snail expression thus promoting apoptosis [58]. Many studies are also focused on examining the effects of combined use of olaparib with liposomal doxorubicin [59, 60], also in the context of limited cardiotoxic effect of doxorubicin with preserved antitumor activity [61, 62]. Combinations with doxorubicin do not exhaust all therapeutic possibilities, because various genotoxic drugs can be combined with PARPis. For example, combinations with platinum derivatives, alkylating compounds, or antimetabolites (all targeting DNA) are successfully used in humans [62]. All these demonstrate the therapeutic potential of employing PARPis and their combinations with synergistic compounds to generate new, molecularly focused therapeutics for malignancies where DNA damage/repair systems play a critical role (Fig. 5).

## Conclusion

Understanding DNA damage disorders in canines will contribute to the development of a reliable method for identifying suitable candidates for PARPis therapy. In the meantime, characterization of the molecular effects of PARPis combinations with other drugs in different cancer types will be the first step to develop new, molecular-based targeted therapeutic strategies in veterinary oncology. The proposed research outputs might also be translated to human medicine. Here, we showed that olaparib may be an effective therapeutic option for canine lymphomas and leukemias. In vitro experiments showed that olaparib inhibits the proliferation of cancerous dog lymphocytes by causing cell DNA damage. The anti-cancer effect of olaparib was visible when the drug was used alone, which indicates DNA repair disorders, but it was particularly pronounced in combination with doxorubicin. Thus, olaparib may be an option for simple oral therapy in canine lymphomas and leukemias and may also be a valuable addition to standard therapeutic protocols.

## Materials and methods

### Cell lines and cell culture

The study involved a panel of 4 different canine lymphoma and leukemia cell lines: CLBL-1 (B-cell lymphoma), GL-1 (B-cell leukemia), CLB70 (B-cell chronic lymphocytic leukemia), and CNK-89 (natural killer-cell lymphoma). The CLBL-1 cell line was obtained from Barbara C. Ruetgen (Institute of Immunology, Department of Pathobiology, University of Veterinary Medicine, Vienna, Austria) [63]; the GL-1 cells were obtained from Yasuhito Fujino and Hajime Tsujimoto (University of Tokyo, Department of Veterinary Internal Medicine) [64], while the CLB70 [65] and CNK-89 [66] cell lines were established in our laboratory. The CLBL-1 and GL-1 cell lines were maintained in the RPMI (Roswell Park Memorial Institute) 1640 medium (Institute of Immunology and Experimental Therapy, Polish Academy of Sciences, Wrocław, Poland), and the CLB70 and CNK-89 cells were cultured in Gibco™ Advanced RPMI 1640 medium (Gibco, Grand Island, New York, USA). All media were supplemented with 2 mM L-glutamine (Sigma Aldrich, Steinheim, Germany), 100 U/mL penicillin, 100 µg/mL streptomycin (Sigma Aldrich), and 10–20% heat-inactivated fetal bovine serum (FBS; Gibco, Grand Island, New York, USA).

### Chemicals and reagents

Olaparib (AZD2281) was obtained from Selleckchem (Cologne, Germany) and dissolved in dimethyl sulfoxide (DMSO) (Sigma Aldrich, Steinheim, Germany) to a final concentration of 50 mM immediately prior to the experiments. Doxorubicin, propidium iodide (PI), 3-[4,5-dimethylthiazol-2-yl]-2,5 diphenyl tetrazolium bromide (MTT), RIPA buffer, and SigmaFAST Protease Inhibitor Cocktail were purchased from Sigma-Aldrich (Steinheim, Germany). Annexin V-FITC was purchased from Immunostep (Salamanca, Spain).

### Cell metabolic activity assay

The metabolic activity of canine cells treated with olaparib was determined using the MTT test. In brief, 1 × 10^5^ cells per well were seeded in a 96-well plate (Thermo Fisher Scientific, Roskilde, Denmark), and olaparib was added at increasing concentrations (0.1, 0.4, 1.2, 3.7, 11.1, and 33.3 µM). The range of concentrations tested was selected based on literature data on the sensitivity of various cell lines to the cytotoxic effects of olaparib and corresponds to the concentrations achievable in vivo [11, 67]. Cells only incubated with DMSO were used as controls. After incubation for 24, 48, 72, and 96 h, 10 µL of MTT solution (5 mg/mL) was added to each well for 4 h. After dissolving the content, the optical density of the wells was measured with a spectrophotometric microplate reader (Spark, Tecan, Singapore) at a reference wavelength of 570 nm. The results were then expressed as the mean of 3 independent experiments (different plates, different days) each one performed in triplicate.

The MTT assay was also used to assess the synergistic effect of olaparib and doxorubicin by comparing cell metabolic activity after treatment with both compounds alone or in combination. To this end, cells seeded at a density of 1.5 × 10^5^/mL (GL-1 cell line) and 3 × 10^5^/mL (CLBL-1) were treated with either olaparib (2.5, 5, and 10 µM for GL-1 and 1.25, 2.5, and 5 µM for CLBL-1 cells) for 96 h or with doxorubicin (25, 50, and 100 nM) for 48 h as single drugs and, for combinatorial treatment, cells were pre-incubated with olaparib for 48 h followed by the addition of doxorubicin for the next 48 h. Olaparib and doxorubicin concentrations were selected based on MTT assay results for single compounds. The data obtained from 3 independent experiments were used to calculate Combination Index (CI) values according to the method established by Chou and Talalay [68] using CompuSyn Software (Informer Technologies, Inc). The Chou-Talalay method is based on a median-effect equation, derived from the mass-action law principle, which allows for determining synergy according to the CI theorem, where CI = 1 means addition, CI < 1 means synergism, and CI > 1 indicates antagonism [68, 69].

### Ki-67 flow cytometry proliferation assay

For this cell proliferation assay, cells were plated at a density of 1 × 10^5^/mL in 96-well plates (TPP, Trasadingen, Switzerland), and incubated for 48 h with two concentrations of olaparib (25 and 50 µM) selected according to MTT test results. After that time, the cells were collected, washed with PBS, labeled using the Ki-67 Proliferation Kit BD Pharmingen (BD Biosciences) according to the manufacturer’s instructions, and then analyzed in a flow cytometer (Cytoflex, Beckman Coulter). Percentages of proliferating (Ki-67 positive) cells were used to calculate the means ± SD for each cell line. The presented results were obtained from 3 independent experiments.

### Western blotting

A total of 5 × 10^6^ cells were rinsed with cold PBS and lysed with RIPA buffer (50 mM Tris-HCl pH 7.5, 100 mM NaCl, 1% NP-40, protease inhibitors set) and incubated for 20 min on ice. Then, after centrifuging at 10,000 rpm at 4 ℃ for 12 min SDS sample buffer was added to clear the supernatants and the samples were boiled at 95 ℃ for 5 min and subjected to SDS-polyacrylamide gel electrophoresis in a 12% gel (BioRad Mini-PROTEAN Tetra Vertical Electrophoresis Cell system, Hercules, USA). After the electrophoresis, the samples were transferred to a nitrocellulose membrane using a BioRad Mini Trans-Blot^®^ Cell for wet transfer and Western Blot enhancer treatment was performed according to manufacturer’s protocol with Pierce™ Western Blot Signal Enhancer (Thermo Scientific). Then, the membranes were blocked with 3% BSA in TBST at room temperature for 1 h. After blocking, the membranes were incubated overnight at 4 ℃ with the following murine monoclonal primary antibodies: anti-γH2A.X clone 9F3 (ab26350; Abcam, Cambridge, United Kingdom), dilution 1:1000 and anti-β-actin clone C4 (sc47 778; Santa Cruz, California, USA), dilution 1:2000. Goat AntiMouse Immunoglobulins/HRP (#P0447; Dako, Agilent; Santa Clara, USA, at 1:20000) was used as secondary antibody. The membranes were incubated with the secondary antibody for 90 min at room temperature. The reaction was developed using Blotting substrate - Pierce™ ECL Western Blotting Substrate (Thermo Scientific) as a substrate. Membrane visualization was performed using ChemiDoc Touch Instruments (exposure: first image, 5 s; last image, 120 s; images, 5; BioRad). For protein expression quantification, Western blot normalization with a single housekeeping protein (β-actin) was performed using Image LabTM software (version 6.1.0; BioRad).

### Next-generation RNA sequencing (NGS)

#### RNA extractions

First, 1 mL of TRIzol Reagent was used to lyse and homogenize the samples and then the material was mixed with 200 µL of chloroform. Samples were centrifuged at 4 ℃ for 20 min at 13,000 rpm following a 2-minute incubation period. After that, the top aqueous phase was gathered, mixed with 70% ice cold ethanol, and moved into a RNeasy spin column. The next steps of the protocol for the RNeasy mini Kit (Qiagen, Hilden, Germany) were carried out according to the manufacturer’s instructions. The Qubit 2.0 Fluorometer (Life Technologies, Carlsbad, CA, USA) and NanoDrop 1000 Spectrophotometer (Thermo Fisher Scientific, Waltham, MA, USA) were used to measure total RNA. The RNA Integrity Number (RIN) value for each sample was greater than 7.

#### RNA-seq library preparation and sequencing

Library preparation and sequencing were performed by Novogene Biotechnology (Cambridge, UK). A total of 8 tagged RNA-seq libraries were prepared and sequenced using a 150 bp strand-specific paired-end strategy on an Illumina Novaseq 6000.

#### Identification of exonic variants from RNA-seq data

RNA-seq data were used to evaluate the exonic mutational profile of CLBL-1 and GL-1 cell lines, the most susceptible and resistant to olaparib, respectively. This evaluation was performed on control, untreated CLBL-1 and GL-1 cells. Nine target genes implicated in DNA damage response (Table 3) were selected. For every cell line, four biological replicates were considered. Raw reads underwent a preliminary quality control with FastQC software (v.0.11.9) before proceeding with the other analyses. Reads were aligned to the reference cDNA sequences retrieved from Ensembl (release 112; GCA_000002285.4; canFam6) (Table 2). Bowtie2 (v.2.5.4) was used to build the indexes and align the reads.

**Table 3:** Target genes selected for the exonic mutational profiling of GL-1 and CLBL-1 cell lines: gene symbol, gene description and ensembl transcript ID

Gene symbol	Gene description	Transcript ID
*BRCA1*	BRCA1 DNA Repair Associated	ENSCAFT00000065506.2
*BRCA2*	BRCA2 DNA Repair Associated	ENSCAFT00000010309.5
*TP53*	Tumor Protein P53	ENSCAFT00000026465.5
*RAD51*	RAD51 Recombinase	ENSCAFT00000073671.2
*ATM*	ATM Serine/Threonine kinase	ENSCAFT00000048574.3
*ATR*	ATR Serine/Threonine Kinase	ENSCAFT00000012571.5
*CHEK1*	Checkpoint Kinase 1	ENSCAFT00000016788.5
*CHEK2*	Checkpoint Kinase 2	ENSCAFT00000018932.5
*STK11*	Serine/Threonine Kinase 11	ENSCAFT00000031055.5

Following alignment, samtools (v.1.19) were used to sort the SAM outputs and convert them to BAM files. An index was also prepared with samtools to visualize them on Integrative Genomic Viewer (IGV)(v.2.18.2). Thanks to IGV visualization, possible single nucleotide variants (SNVs) and insertions-deletions mutations (INDELs) were recognized, and each variant was checked for possible strand biases. The corresponding CDS, and the encoded protein position were retrieved from the Ensembl Genome Browser.

To check for the possible biological consequences of SNVs, VEP and Fido-SNP platforms were used. The prediction of the biological effect of VEP is based on SIFT score [70], while Fido-SNP is a binary classifier based on the Gradient Boosting algorithm [71]. Both tools allow for the prediction of the pathogenicity of SNVs in both coding and non-coding regions of the dog genome. SIFT-VEP scores only missense SNVs, while Fido-SNP scores both synonymous and non-synonymous SNVs. For INDELs that couldn’t be evaluated with the previous software, the nucleotide sequence was converted into the corresponding amino acid sequence checking the possible effect of mutations on the reading frame using EMBOSS Transeq.

### Statistical analysis

All data are shown as means with SD. Statistical differences were analyzed using one-way analysis of variance (ANOVA) followed by the Dunnett’s multiple comparison test (Ki-67 expression marker analysis, DNA damage induction assessment). Statistical analysis was performed using either STATISTICA software version 13.3 (TIBCO Software Inc., Palo Alto, CA, USA) or GraphPad Prism v.9 (GraphPad Software, San Diego, CA, USA). The results were considered significant at *P* <.05.

## Electronic supplementary material

Below is the link to the electronic supplementary material.

Supplementary Material 1

Supplementary Material 2

Supplementary Material 3

Supplementary Material 4

Supplementary Material 5

Supplementary Material 6

Supplementary Material 7

Supplementary Material 8

Supplementary Material 9

Supplementary Material 10

## Data Availability

Raw Illumina sequencing data have been deposited in GenBank (SRA) under the BioProject accession PRJNA1242827. Additional supporting information can be found online in the Supporting Information section at the end of this article (Supplementary Table 1 with complete list of variants identified in DNA-damage response genes of CLBL-1 and GL-1 cell lines).

## Abbreviations

AICM: Anthracycline-induced Cardiomyopathy
ANOVA: Analysis of Variance
ATM: Ataxia Telangiectasia Mutated
ATR: Ataxia Telangiectasia and Rad3-related
BRCA1: Breast Cancer Associated 1
BRCA2: Breast Cancer Associated 2
AKD: Cyclin-dependent Kinase
CDK12: Cyclin-dependent Kinase 12
CDS: Coding Sequence
CI: Combination Index
CHK1: Checkpoint kinase 1
CHK2: Checkpoint kinase 2
CLB70: Canine B-cell Chronic Lymphocytic Leukemia Cell Line
CLBL-1: Canine B-cell Lymphoma Cell Line
CNK-89: Canine Natural Killer-cell Lymphoma Cell Line
DCM: dilated cardiomyopathy
DMSO: Dimethyl Sulfoxide
DSBs: Double-Strand Breaks
FANCL: Fanconi Anemia Complementation Group L
FBS: Fetal Bovine Serum
FITC: Fluorescein Isothiocyanate
γH2AX: Phosphorylated Histone H2AX
GL-1: B-cell Leukemia Cell Line
HE: Heterozygous
HO: Homozygous
HR: Homologous Recombination
IC_50_: Half Maximal Inhibitory Concentration
IGV: Integrative Genomic Viewer
INDELs: Insertions or Deletions
ITDs: Internal Tandem Duplications
MAPK12: Mitogen-Activated Protein Kinase 12
MCTs: Mast Cell Tumors
MTT: 3-[4,5-dimethylthiazol-2-yl]-2,5-diphenyl tetrazolium bromide
NGS: Next-Generation Sequencing
PARP: Poly(ADP-ribose) Polymerase
PARPi: PARP Inhibitors
PI: Propidium Iodide
PI3K: Phosphoinositide 3-Kinases
PLK3: Polo-Like Kinase 3
PNKP: Polynucleotide Kinase-Phosphatase
RAD51: RAD51 Recombinase
SIFT: Sorting Intolerant From Tolerant
SSBs: Single-Strand Breaks
SNVs: Single-nucleotide Variants
STK22C: Serine/Threonine-Protein Kinase 22 C
STK36: Serine/Threonine Kinase 36
TP53: Tumor Protein P53
