# Supplementary material for: PARP inhibitor olaparib induces DNA damage and acts as a drug sensitizer in an in vitro model of canine hematopoietic cancer
Source: BMC Vet Res. 2025 Jul 5;21:439. doi: 10.1186/s12917-025-04880-z (PMC12228278; doi:10.1186/s12917-025-04880-z)
Supplement: Supplementary file 2 — Supplementary Material 2 [file 12917_2025_4880_MOESM2_ESM.docx]

**File: Ola farmakologia 2024-11-28 15h06m04s**

Cell lines: CLBL-1, GL-1

From left to right:
lane 1; CLBL-1 0 µM, lane 2; CLBL-1 25 µM, lane 3; CLBL-1 50 µM, lane 4; GL-1 0 µM, lane 5; GL-1 25 µM, lane 6; GL-1 50 µM


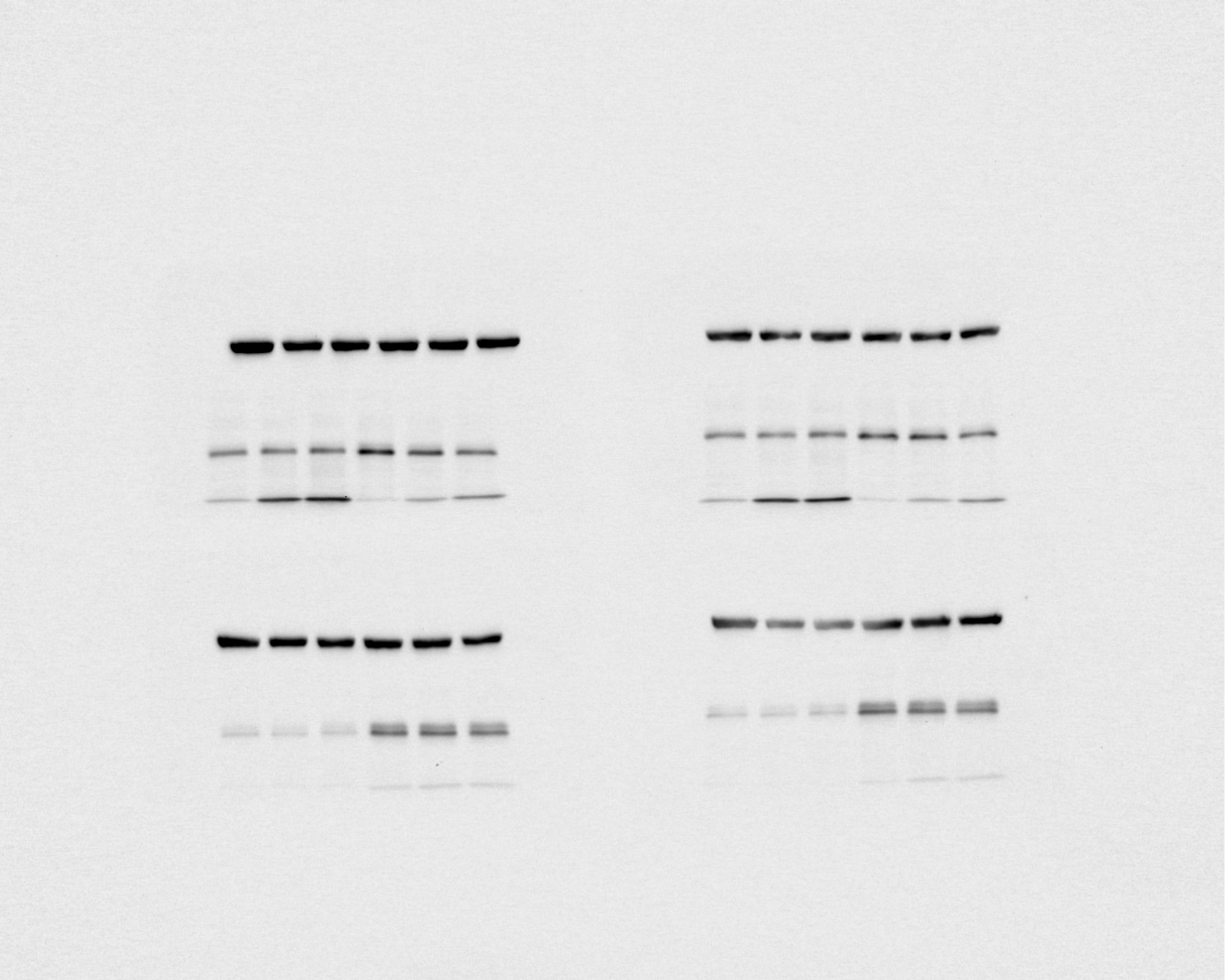


1 2 3 4 5 6

1 2 3 4 5 6

1 2 3 4 5 6

1 2 3 4 5 6

**File: Ola farmakologia 2024-11-29 14h14m52s**

Cell lines: CLBL-1, GL-1

From left to right:
lane 1; CLBL-1 0 µM, lane 2; CLBL-1 25 µM, lane 3; CLBL-1 50 µM, lane 4; GL-1 0 µM, lane 5; GL-1 25 µM, lane 6; GL-1 50 µM


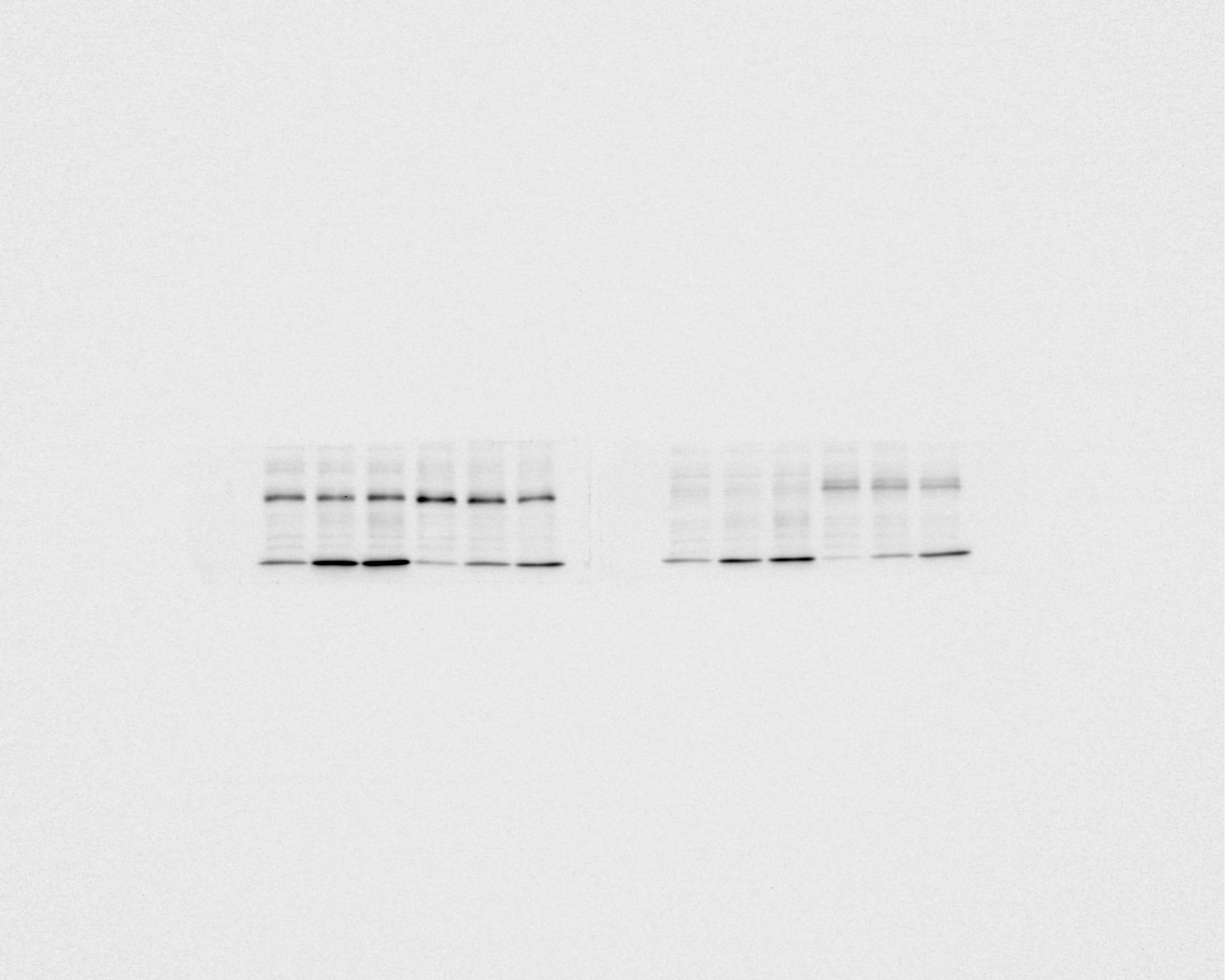


1 2 3 4 5 6

1 2 3 4 5 6

**File: Ola farmakologia 2024-12-05 14h34m47s**

Cell lines: CNK-89, CLB70

From left to right:
lane 1; CNK-89 0 µM, lane 2; CNK-89 25 µM, lane 3; CNK-89 50 µM, lane 4; CLB70 0 µM, lane 5; CLB70 25 µM, lane 6; CLB70 50 µM


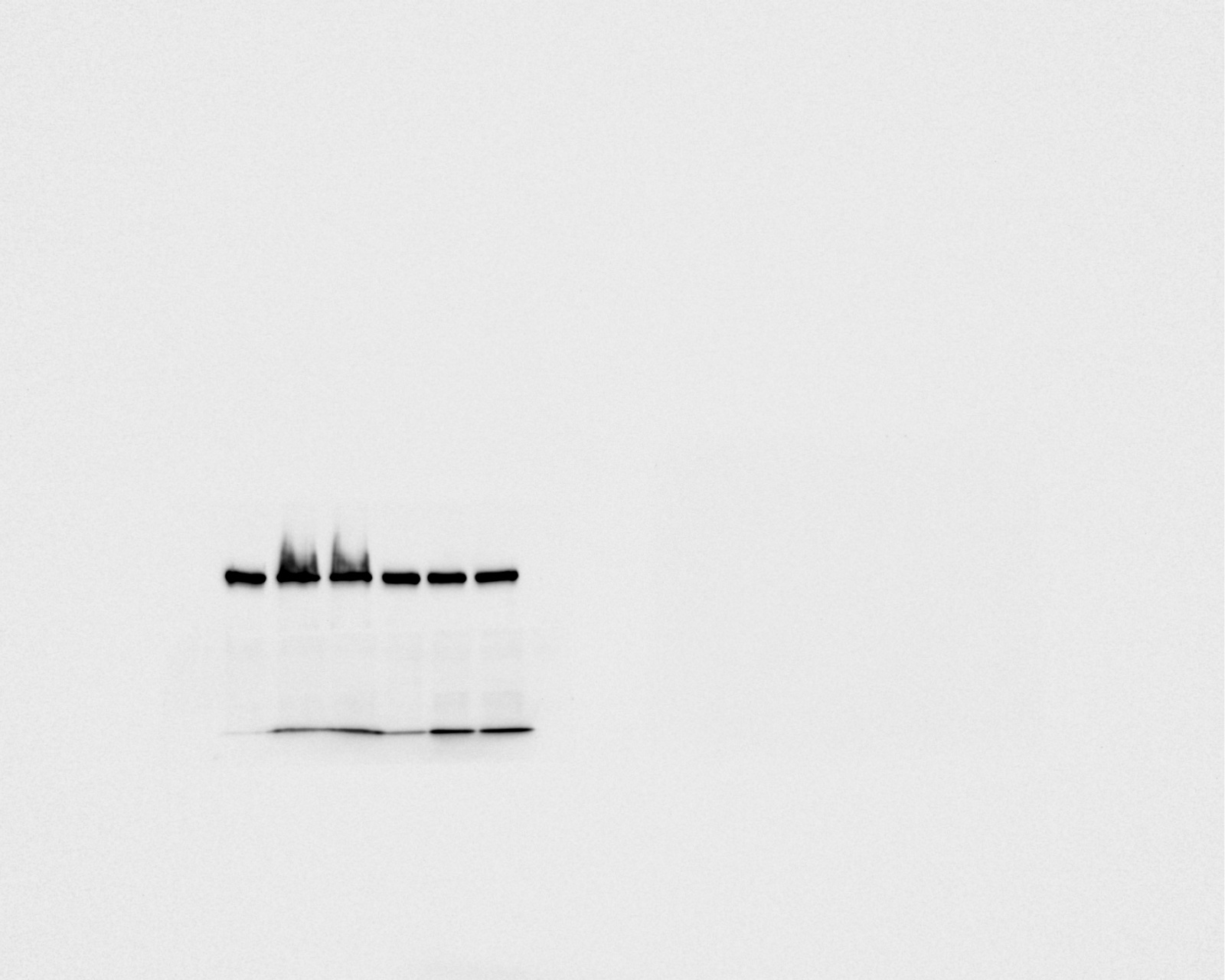


1 2 3 4 5 6

**File: Ola farmakologia 2024-12-09 14h20m14s**

Cell lines: CNK-89, CLB70 **(On this image only lower membranes belongs to CNK-89 and CLB70 cell lines**)

From left to right:
lane 1; CNK-89 0 µM, lane 2; CNK-89 25 µM, lane 3; CNK-89 50 µM, lane 4; CLB70 0 µM, lane 5; CLB70 25 µM, lane 6; CLB70 50 µM


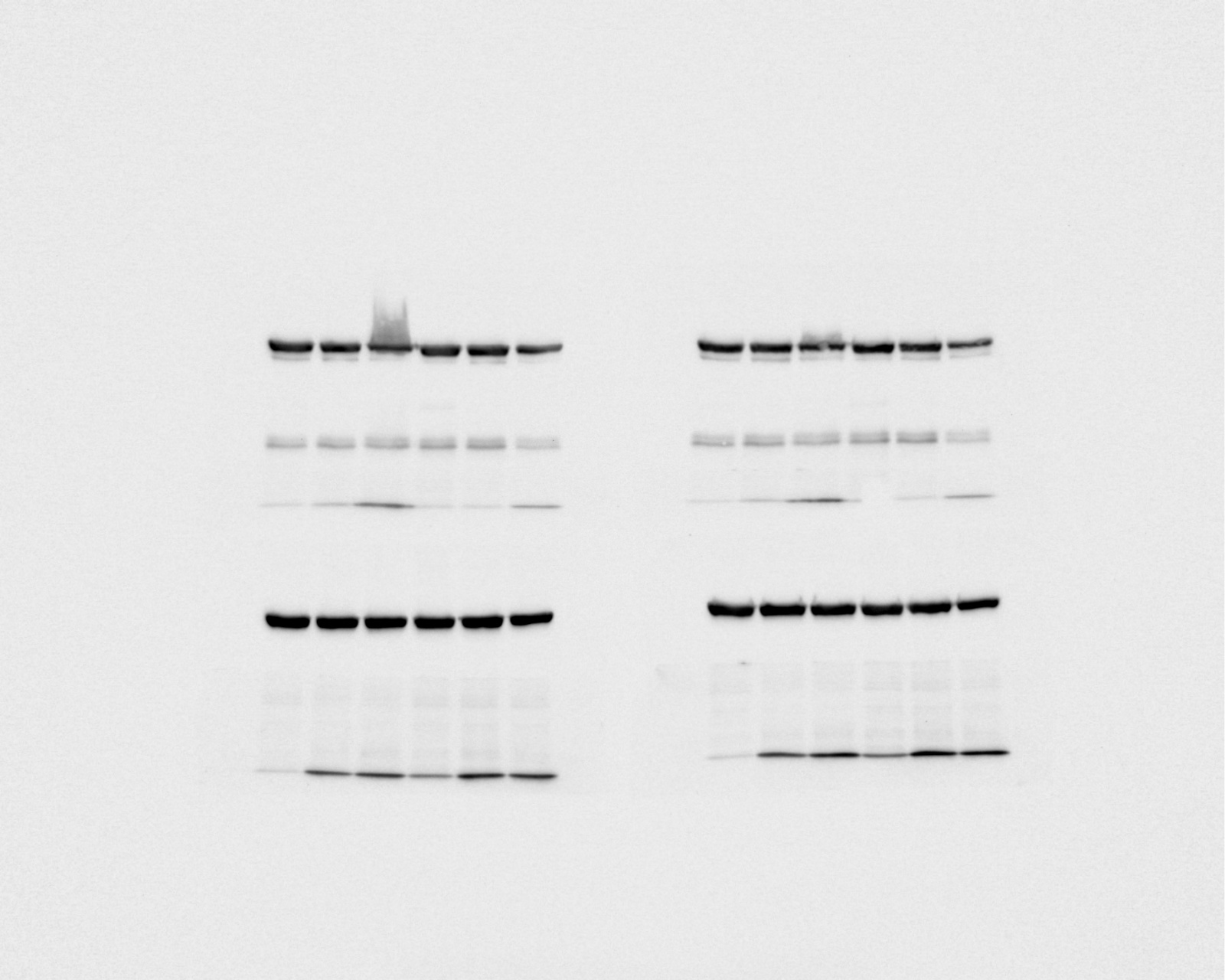


1 2 3 4 5 6

1 2 3 4 5 6
