# Supplementary figures and images for: PARP inhibitor olaparib induces DNA damage and acts as a drug sensitizer in an in vitro model of canine hematopoietic cancer
Source: BMC Vet Res. 2025 Jul 5;21:439. doi: 10.1186/s12917-025-04880-z (PMC12228278; doi:10.1186/s12917-025-04880-z)

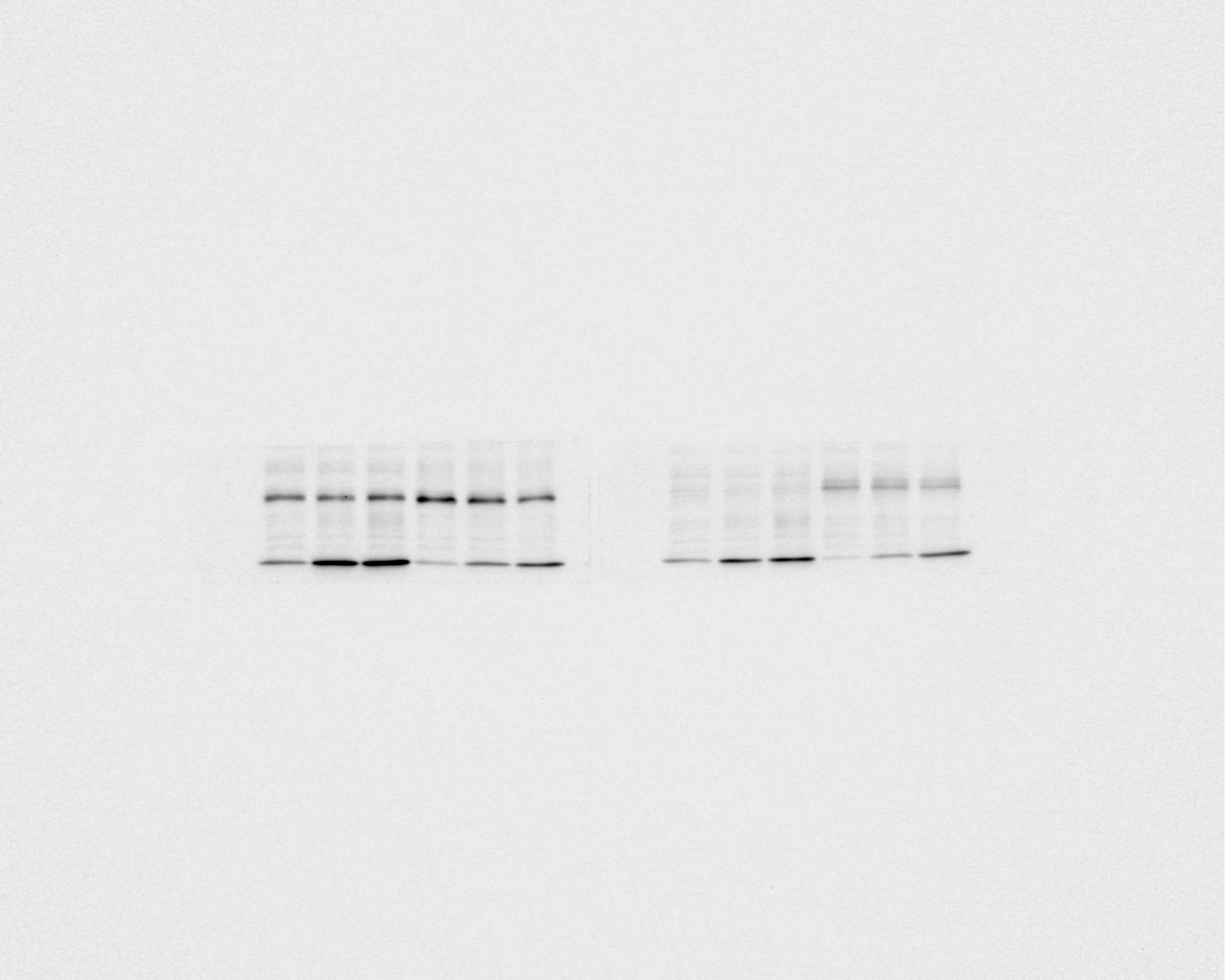

Supplement: Supplementary file 3 — Supplementary Material 3 [file 12917_2025_4880_MOESM3_ESM.jpg]

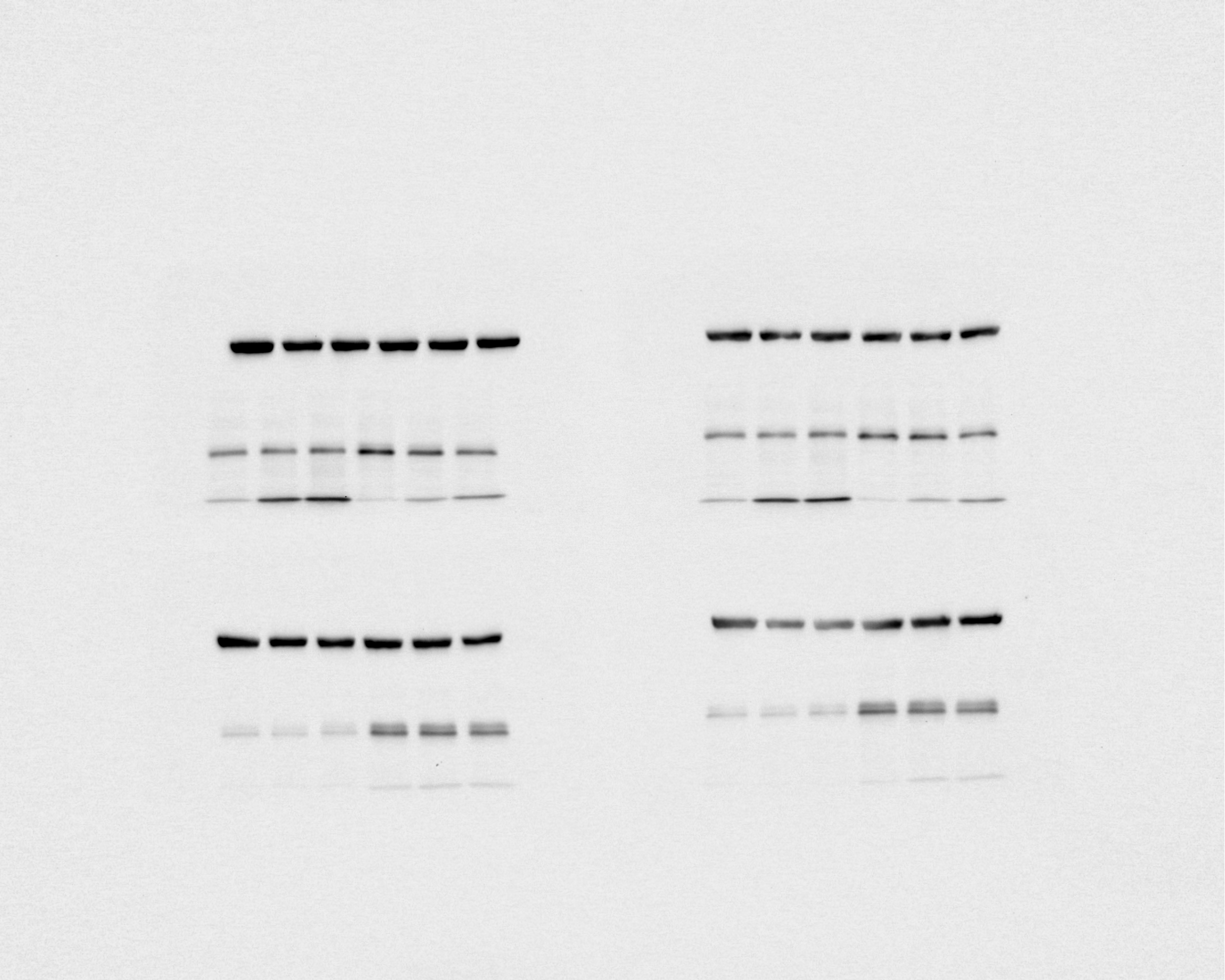

Supplement: Supplementary file 4 — Supplementary Material 4 [file 12917_2025_4880_MOESM4_ESM.jpg]

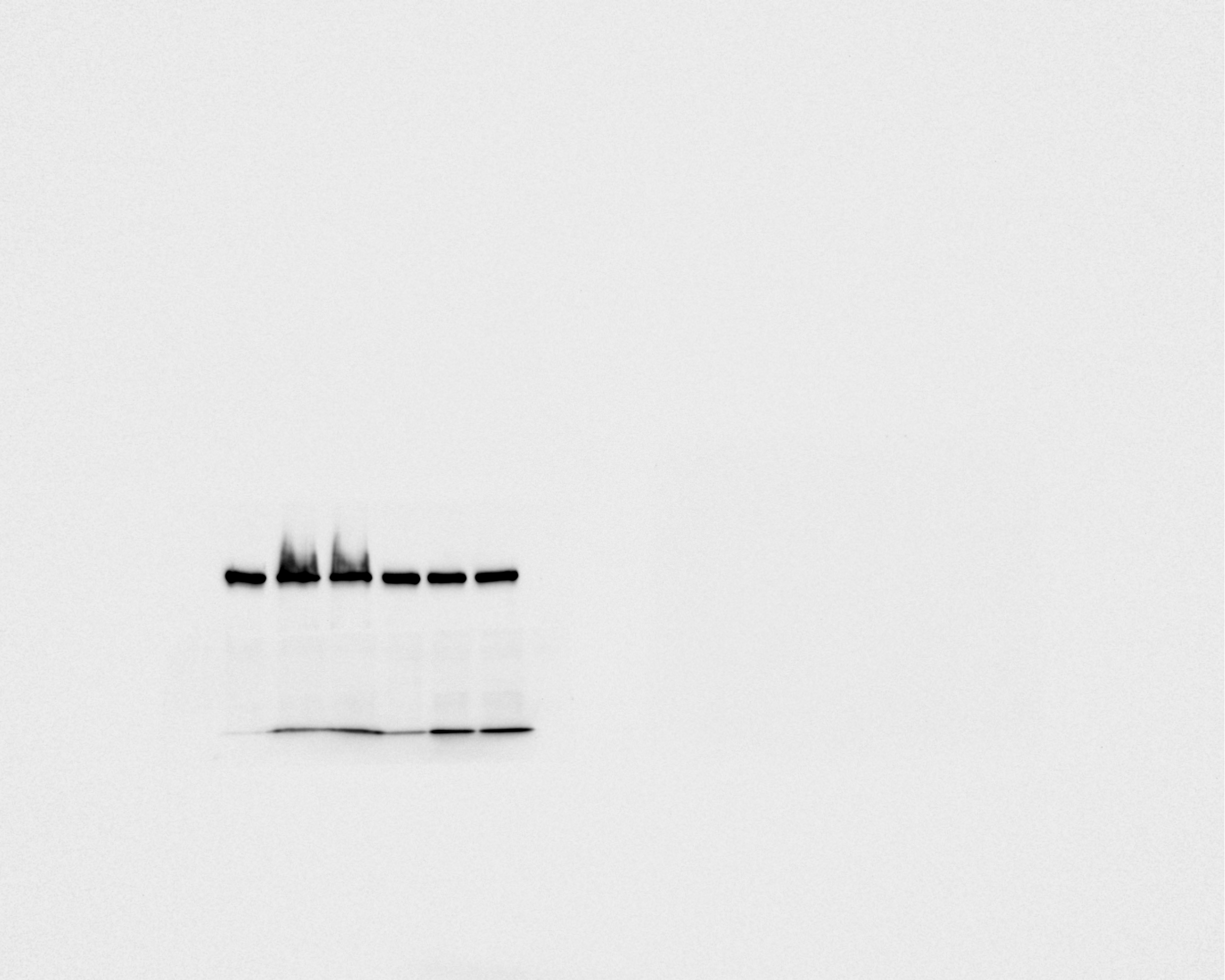

Supplement: Supplementary file 5 — Supplementary Material 5 [file 12917_2025_4880_MOESM5_ESM.jpg]

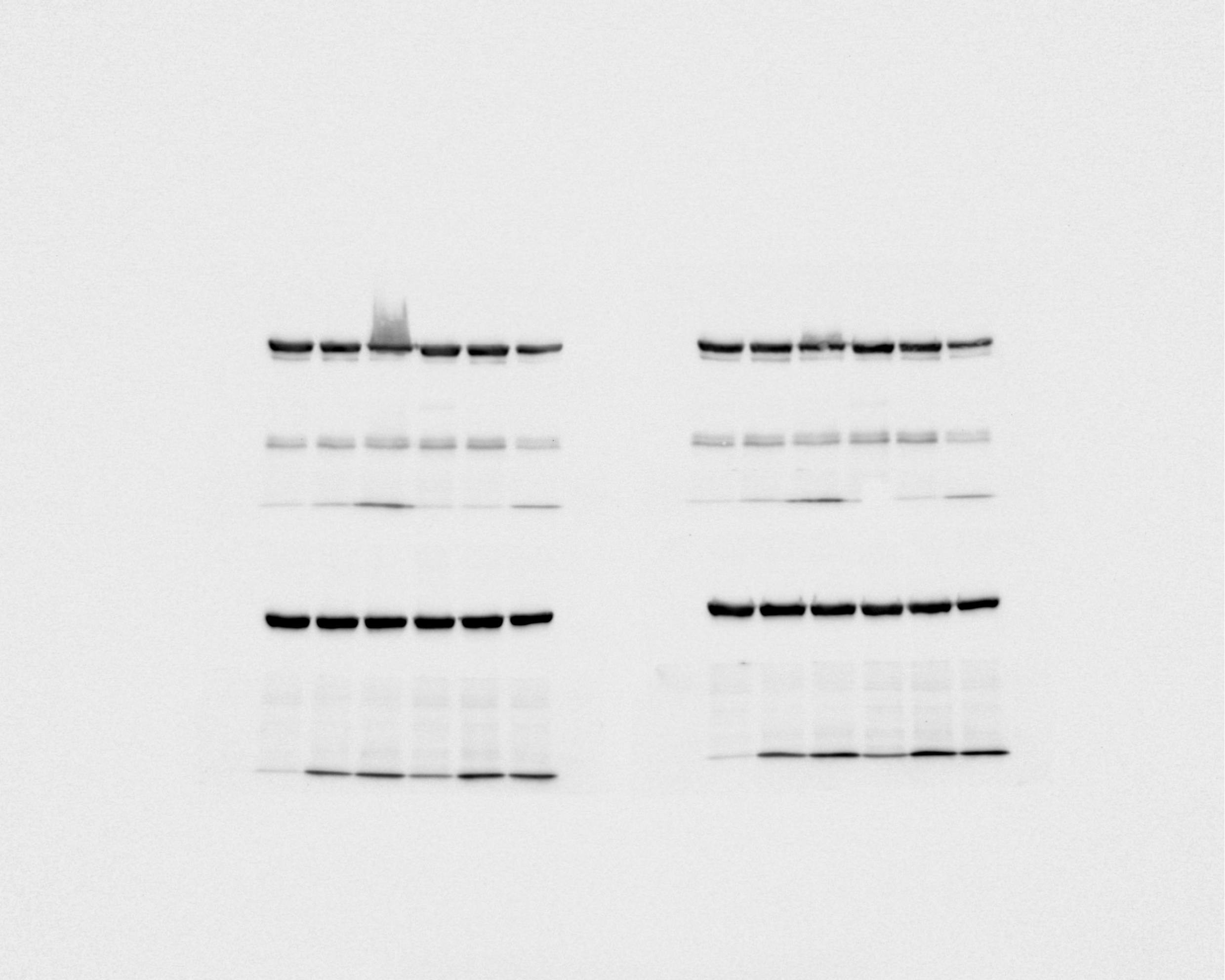

Supplement: Supplementary file 6 — Supplementary Material 6 [file 12917_2025_4880_MOESM6_ESM.jpg]
